# Making the invisible, visible: a cross-sectional study of late presentation to HIV/AIDS services among men who have sex with men from a large urban center of Brazil

**DOI:** 10.1186/1471-2458-14-1313

**Published:** 2014-12-22

**Authors:** Sarah MacCarthy, Sandra Brignol, Manasa Reddy, Amy Nunn, Ines Dourado

**Affiliations:** Rand Corporation, 1776 Main Street, Santa Monica, CA 90407 USA; Instituto de Saúde Coletiva/Universidade Federal da Bahia, Universitário do Canela, Av. Basílio da Gama, s.n. Campus, Salvador, Bahia CEP: 40.110-040 Brasil; Alpert Medical School of Brown University and The Miriam Hospital, 164 Summit Avenue, Providence, RI 02906 USA

**Keywords:** HIV/AIDS, Late presentation, Men who have sex with men

## Abstract

**Background:**

Late presentation to testing, treatment and continued care has detrimental impacts on the health of HIV-positive individuals as well as their sexual partners’ health. Men who have sex with men (MSM) experience disproportionately high rates of HIV both globally and in Brazil. However, the factors that inhibit linkage to care among MSM remain unclear.

**Methods:**

We conducted a cross-sectional study of HIV-positive MSM (n = 740) enrolled in HIV/AIDS services in a large urban center of Brazil from August 2010 to June 2011. Descriptive, bivariate and multivariate statistics were conducted using STATA 12 to examine the relationship between a range of variables and late presentation, defined as having a first CD4 count <350 cells/mm^3^.

**Results:**

Within the sample, the prevalence of LP was 63.1%. Men who self-identified as heterosexual (AOR 1.54 and 95% CI 1.08 - 2.20) compared to men who self-identified as homosexual and bisexual were at increased odds of late presentation. Additionally, men age 30 and older (AOR 1.56, 95% CI 1.01 – 2.43) compared to individuals age 18–29 experienced increased odds of late presentation among MSM.

**Conclusions:**

The prevalence of LP in this population was higher than noted in the global literature on LP among MSM. Heterosexual men and older age individuals experienced substantial barriers to HIV care. The stigma around same-sex behaviors and the current focus of HIV prevention and treatment campaigns on younger age individuals may limit patients’ and providers’ awareness of the risk for HIV and access to available services. In addition to addressing HIV-specific barriers to care, developing effective strategies to reduce late presentation in Brazil will require addressing social factors - such as stigma against diverse sexualities - to concretely identify and eliminate barriers to available services. Only in so doing can we make currently invisible people, visible.

## Background

Substantial research among men who have sex with men (MSM) has documented severe disparities in HIV rates and in access to HIV services. A recent meta-analysis and systematic review estimated pooled HIV prevalence rates ranging from 3% in the Middle East to 25% in the Caribbean [1]. In Brazil, the first country to provide free and universal treatment to people living with HIV [2], a large national study of 3859 MSM from 10 cities reported that the HIV prevalence ranged from 5 to 24% with a pooled prevalence of 14% among all cities [3]. In Salvador, the third largest and one of Brazil’s poorest cities, the prevalence of HIV among MSM was 7% (3%-10%) [3].

Studies have also documented low levels of MSM accessing HIV services. Global weighted estimates based on UNGASS country progress reports found HIV prevention programs reached only one-third of MSM in low- and middle-income countries [4]. One- to two-thirds of MSM surveyed in middle-income countries reported never having been tested for HIV [4–8]. In qualitative interviews, men cited fear of stigma, low risk perception, or lack of available services as the largest barriers to testing [5, 9, 10]. In particular, the disparities among young black men are substantial: a global study by Millett and colleagues found that despite reporting lower levels of risk-taking behavior compared to other racial and ethnic groups, HIV-positive black MSM were less likely to start antiretroviral therapy (ARVs) than men of other races and ethnicities [11].

The importance of timely access to HIV services, including testing, linkage to treatment and continued retention in care, is increasingly recognized [12] at both the individual and population levels. Specifically, studies have shown that late presentation (LP) to these services has severe consequences for the morbidity and mortality of individuals living with HIV [13, 14] and can also increase the risk of transmission to others [13]. For example a recent analysis of national data in the United States found that HIV-positive MSM who were unaware of their HIV-status were also significantly more likely than other MSM (HIV-negative men and HIV-positive men who were aware of their diagnosis) to engage in unprotected discordant anal sex [15]. Further, there are substantial economic [16] and psychological [17] costs associated with late presentation to HIV/AIDS care. To date, little is known about the rates of LP among MSM: a study from Germany and Spain reported between 45% and 48% of MSM presented late to HIV/AIDS services respectively [18, 19]. However these studies simply reported the prevalence and did not further disaggregate the data to identify the factors associated with LP among MSM. Further, while access to ARVs is increasingly available, studies documenting late presentation to services in low- and middle-income country contexts are lacking. Therefore we examined the prevalence of LP and the factors associated with LP among MSM in Brazil.

## Methods

We conducted a cross-sectional study and collected data among HIV-infected men in Northeast Brazil. All participants were HIV-infected, age 18 years or older, and enrolled for clinical care for the first time at one of three main health facilities in Salvador, Brazil from August 2010 to June 2011 (n = 740).

### Setting

The facilities included the following: the HIV/AIDS specialty care center and two large hospitals providing general and HIV/AIDS outpatient care. Since 1997, the Brazilian government has provided HIV/AIDS care and treatment free of charge at all facilities belonging to the Brazilian National Public Health System [20]. The facilities were located in the city of Salvador, capital of the northeastern state in Bahia. Salvador is the third most populous city in the country with approximately 2.7 million people and is also one of the poorest cities in the country [21] Salvador was the main port of entry for the Brazilian slave trade for more than 200 years and is home to a large afro-descendent population.

### Procedures

Study staff attended the HIV specialty care center and the HIV outpatient care at the two hospitals daily. A list of scheduled patients was provided to the study team beforehand. Refusal to participate was minimal (less than 5%). Inclusion criteria were age 18 or older and laboratory confirmed diagnosis of HIV infection. Exclusion criteria were receiving initial HIV/AIDS care at another health facility from where the interview was being conducted and diagnosis with mental health disorders. All patients were counseled that participation in the study entailed responding to our survey and allowing access to their laboratory data. Further, the participants were asked to sign a consent form. The interviews were individually conducted in a private space at the facility with trained research staff. Responses were recorded digitally using a palm pilot. The interview included questions related to socio-demographic characteristics, access to HIV/AIDS services, as well as sexual and other behaviors associated with LP in the peer-reviewed literature. No financial incentives were provided.

### Study participants

**Figure 1:**
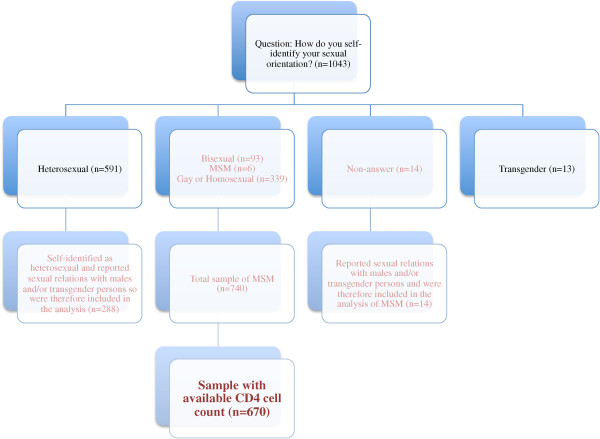
**Defining the sample of MSM based on reported sexual behavior (n = 670).**

Special precautions were taken to define the study population. Since the term “MSM” was originally created by the public health community to focus on the sexual behaviors related to elevated risk of HIV [22], the authors developed a strategy to ensure the analyses compared individuals based on similar sexual behaviors, not self-reported sexual orientation, which may be recorded differently based on a variety of factors. As described in Figure 1, the following criteria were used to identify MSM: 1) self-identified as homosexual, bisexual, or MSM; and/or 2) reported penetrative (insertive or receptive anal sex) or oral sex with other men within the 12 months preceding the interview; and/or 3) reported having a main or non-main male or a transgender partner within the 12 months preceding the interview.

### Primary outcome of interest

The LP outcome was based on a definition published in 2011 by the European Late Presenter Consensus working group which established LP as “presenting for care with a CD4 count below 350 cells/mm^3^ or presenting with an AIDS defining event, regardless of the CD4 cell count” [23]. Though this outcome does not distinguish whether or not individuals are late to testing or subsequent linkage to care [24], the consensus definition still provides general insight regarding individuals presenting to care late [23]. For our analysis, LP was restricted to individuals with an available CD4 cell count, as there was no clinical data available to identify individuals defined as AIDS cases based on clinical symptoms. Among the sample population, 10% (n = 70) of men did not have their CD4 cell count available; therefore 670 were included in this analysis. Descriptive statistics on individuals who were excluded showed no significant differences between this group and the sample population. In Salvador, individuals can only have a CD4 test conducted once they have enrolled in HIV care therefore LP in our sample represents the level of disease progression once people have tested positive for HIV and successfully linked to care.

To complete and validate data on first CD4 cell count, information was reviewed by trained research assistants from hand written clinical records and from SISCEL (Laboratory Test Control System of the Brazilian National CD4+/CD8+ T Lymphocyte Count and Viral Load Network) which contains information on lymphocyte count CD4/CD8 and viral load. This system is organized by patient identifier code, and the database is powered by a nationwide laboratory network.

### Key variables of interest

Key variables of interest are highlighted in the descriptive statistics (Table 1). A variety of key variables were explored. A range of options were given for self-reported sexual orientation, however the only responses selected included MSM, homosexual, bisexual, and heterosexual. In Brazil, race and ethnicity is commonly referred to as ‘color’ to reference the phenotype (physical appearance) and not one’s ancestry (origin) [25] and therefore four categories were constructed: 1-brown; 2-black; 3-white; 4- yellow, indigenous and other. Age was dichotomized based on the traditional cut point for young MSM age (18–29 years vs. 30 and older). This cut point was used because a global meta analysis on HIV among MSM suggested 30 was an important marker to distinguish between young and older MSM, therefore we used this same age structure to be consistent with the peer-reviewed literature [11]. The remaining socio-demographic variables were dichotomized: individual income (receiving minimum wage of $510 Brazilian Reis per month = $328.11 USD per month or less vs. receiving above it); employment (individuals formally and informally employed vs. unemployed); Years of schooling (receiving the minimum eight years of schooling vs. receiving more); and living location (living in the greater metropolitan region of Salvador vs. those living outside of Salvador). As for other characteristics of interest, history of smoking (smoking ever vs. never reported smoking); history of drug use (drug use ever vs. never reported drug use); and history of STI (never had a diagnosed STI vs. diagnosed STI at least one time). Variables related to potential stigma and discrimination were experience with forced sex (no vs. yes) and HIV-related discrimination (often, sometimes, and few times vs. never). We investigated the number of sexual partners in the last 12 months (comparing individuals reporting one or less to individuals reporting more) and whether participants held private health insurance (no vs. yes).

**Table 1:** **Descriptive statistics of MSM receiving HIV/AIDS care in Salvador, Brazil**

Variables	n	%
*Prevalence of late presentation*		
Less than 350 cells per mm^3^	423	63.1
Equal or more than 350 cells per mm^3^	247	36.9
*Self-reported sexual orientation*		
Homosexual	345	47.5
Bisexual	93	12.8
Heterosexual	288	39.7
Skin color*		
White	67	9.1
Yellow | Indigenous | Other	70	9.5
Brown	320	43.4
Black	280	38.0
*Age*		
18– 29	90	13.4
30 and older	580	86.6
Income**		
More than minimum wage	580	78.4
Minimum wage or less	160	21.6
*Employment*		
Employed	395	53.5
Unemployed	344	46.5
*Years of schooling*		
More than 8 years of education	470	63.5
8 years or less of education	270	36.5
*Living location*		
Salvador	583	78.7
Metropolitan region of Salvador	157	21.2
*History of smoking*		
No	384	52.0
Yes	355	48.0
*History of drug use*		
No	544	73.5
Yes - At least one time	196	26.5
*History of a sexually transmitted infection*		
Never	385	52.0
At least one time	351	48.0
*Experience with forced sex*		
Never	661	89.3
Yes - at least one time	79	10.7
*HIV related discrimination*		
Never	561	75.9
Yes - at least one time	178	24.1
*Number of sexual partners in the last 12 months*		
Less than one	241	35.0
More than one	447	65.0
*Owns private health insurance*		
Yes	117	17.5
No	553	82.5

*Race is commonly referred to as *cor* or ‘color,’ and references the phenotype (physical appearance) and not one’s ancestry (origin).

**Minimum wage of $510 BR per month = $328.11 USD per month as established by the Brazilian government at the time data was collected.

### Analysis

The model building process followed these steps: 1) A review of the literature was conducted to identify factors consistently highlighted in studies on LP. 2) Bivariate analyses were conducted to identify additional variables for inclusion in the multivariate analysis that were not already highlighted in the literature (Table 2). 3) Further diagnostic tests were conducted to determine if variables were correlated. For example, though individual income, employment and years of school all had a p-value < 0.20, a diagnostic test suggested that all three variables were correlated and therefore only income was included in the model given that it reported the tightest confidence interval. As a result of the bivariate analysis, history of STI, experience with forced sex, and smoking were included in the final model. 4) The logistic regression analyses started with a saturated model, and variables were progressively removed to identify the best model and adjusted odds ratios (AOR) and 95% confidence intervals (CI) were estimated. Additional tests examining outliers were conducted and the goodness of fit chi-square Pearson test was used to evaluate the final models. The analyses were completed using STATA^®^ (Statistics Data Analysis, version 12.0).

**Table 2:** **Bivariate statistics including the number, proportion, odds ratio (OR) and 95% confidence interval (CI) of late presentation among MSM receiving HIV/AIDS care in Salvador, Brazil**

Variables		Late presenters		
	n = 423	(%)	OR	95% CI
*Self-reported sexual orientation*				
Homosexual and bisexual	235	58.8	1.00	-
Heterosexual	179	69.4	1.59	1.14 - 2.22
Skin Color*				
White | Yellow | Indigenous | Other	43	65.0	1.00	-
Brown	186	62.6	0.90	0.58 - 1.40
Black	156	63.2	0.92	0.59 - 1.45
*Age*				
18 – 29	47	52.2	1.00	-
30 years or older	376	64.8	1.69	1.08 - 2.64
Individual Income**				
> Minimum wage	321	61.3	1.00	-
≤ Minimum wage	102	69.9	1.48	1.00 - 2.18
*Employment*				
Employed	209	59.0	1.00	-
Unemployed	213	67.9	1.47	1.07 - 2.02
*Years of schooling*				
>8 years	165	68.8	1.00	-
≤8 years	258	60.0	1.47	1.05 - 2.05
*History of smoking*				
No	210	60.5	1.00	-
Yes	213	66.2	1.27	0.93 - 1.75
*History of drug use*				
No	317	64.4	1.00	-
Yes	106	59.6	0.81	0.57 - 1.16
*History of STI*				
Never	232	66.3	1.00	-
≥ Once	189	59.6	0.75	0.55 - 1.03
*Experience with forced sex*				
Never	597	64.3	1.00	-
At least once	73	53.4	0.63	0.40 - 1.04
*HIV related discrimination*				
Never	311	62.1	1.00	
Yes - at least one time	112	66.7	1.22	0.85-1.77
*Owns private health insurance*				
No	64	54.7	1.00	
Yes	359	64.9	1.53	1.02 - 2.30

*Race is commonly referred to as *cor* or ‘color,’ and references the phenotype (physical appearance) and not one’s ancestry (origin).

**Minimum wage of $510 BR per month = $328.11 USD per month as established by the Brazilian government at the time data was collected.

This study was approved by the Ethics and Research Committee of the State Health Department of Bahia, on November 5, 2009 (official record 073/2009) and written informed consent was given by all participants.

## Results

The prevalence of LP was 63.1% (95% CI: 59.5%-66.8%) among MSM and full descriptive statistics are included in Table 1. With respect to self-identified sexual orientation, 47.5% of respondents self-identified as homosexual, 12.8% as bisexual, and 39.7% as heterosexual. In our sample 43.4% self-identified as “brown,” 38.0% as “black,” 9.1% as “white,” and 9.5% as “yellow, indigenous, or other.” ‘Young MSM’, or those 18–29 years of age constituted 13.4% of the sample. Just over half (53.5%) were employed and 78.4% reported an income above official minimum wage. More than half of participants (63.5%) had completed more than eight years of education. At the time of the survey, most respondents (78.7%) lived in the city of Salvador. Further, 73.5% reported no history of drug use, 52.0% reported no history of STIs, 10.7% reported a history of forced sex, and 24.1% reported experiencing discrimination based on their HIV status at least one time. Approximately 65.0% of all respondents reported having more than one sexual partner in the past 12 months. Finally, with respect to the HIV services provided, a majority of participants (82.5%) did not own private health insurance.

The multivariate regression analysis found increased odds for LP among individuals who self-identified as heterosexual (AOR 1.54, 95% CI 1.08-2.20) compared to individuals who self-identified as homosexual or bisexual. In addition, individuals age 30 and older (AOR 1.64, 95% CI 1.02-2.64) compared to individuals age 18–29 experienced increased odds of LP. These results are shown in Table 3.

**Table 3:** **Adjusted odds ratios for the association between late presentation and study variables among MSM receiving HIV/AIDS care in Salvador, Brazil**

Variables	Adjusted OR	95% CI
*Self-reported sexual orientation*		
Homosexual and bisexual	1.00	
Heterosexual	1.54	1.08 - 2.20
*Age*		
18-29 years	1.00	
30 years or older	1.64	1.02 - 2.64
Skin Color*		
White | Yellow | Indigenous | Other	1.00	
Brown	0.82	0.52 - 1.30
Black	0.92	0.57 - 1.49
Income**		
Above minimum wage	1.00	
Minimum wage or less	1.47	0.96 - 2.23
*History of smoking*		
No	1.00	
Yes	1.34	0.94 - 1.89
*History of drug use*		
No	1.00	
Yes	0.69	0.45 - 1.03
*History of a STI*		
Never	1.00	
At least one time	0.73	0.52 - 1.01
*Experience with forced sex*		
Never	1.00	
At least one time	0.70	0.41 - 1.18
*Owns private health insurance*		
No	1.00	
Yes	0.71	0.46 - 1.09

*Race is commonly referred to as *cor* or ‘color,’ and references the phenotype (physical appearance) and not one’s ancestry (origin).

**Minimum wage of $510 BR per month = $328.11 USD per month as established by the Brazilian government at the time data was collected.

## Discussion

The prevalence of LP (63.1%) in this population was higher than noted in the global literature on LP among MSM. For example, studies using the same CD4 threshold of 350 cells/mm^3^ to define LP found between 45% and 48% of MSM presented late to HIV/AIDS services [18, 19]. Further, studies among the general population report between 38% and 63% presented late to HIV/AIDS services [18, 19, 26–33], and a study in Brazil reported 44% of the population presented late [34]. The known risk of transmission with unprotected anal intercourse often contributes to MSM being at the center of prevention and treatment efforts. However, our results suggest that despite this, MSM from our sample relay a prevalence of LP similar to studies among the general population [35]. Thus, even though treatment for HIV has been free and universally available in Brazil since 1996, other factors continue to provide barriers to MSM accessing care.

Our analysis showed that MSM who self-identified as heterosexual experienced increased odds of LP. Many US studies have focused on African American men, and to a lesser extent Latino men, living on the “down low” [36], a colloquial term used to describe self-identified heterosexual men reporting sex with other men [37–39]. A consistent limitation across many of these studies, however, was that minimal attention was given to how the dissonance between self-reported sexual orientation and sexual behavior may be associated with more restricted access to care. Recently, however, quantitative surveys of MSM in Canada, the United States, and Australia have found those who did not disclose same-sex sexual activity to their primary care provider were less likely to have been tested for HIV [40, 41] or vaccinated against Hepatitis A and B [42]. Additionally, the aforementioned study on late presentation among the general population of Brazil found men who self-identified as heterosexual were more likely to present late to HIV/AIDS services, though further analysis to explore this relationship by sexual behaviors was not conducted [34]. Thus, our finding from Brazil contributes to the growing base of evidence that suggests challenges around disclosure of same-sex sexual behavior may be associated with delayed presentation to care.

What remains unclear, however, is the reason as to why men who report discordant sexual orientations and sexual behaviors are at increased odds for late presentation to services. Our results suggest that in Brazil, despite being a country often heralded for its success in addressing LGBT health more generally [43] and especially in the context of HIV [2], a strong undercurrent of homophobia persists [44] and may prevent individuals from accessing available services. Studies from the US and Australia have suggested that compared to their gay-identified counterparts, non-gay identified men may have lower perceived risk of HIV or less knowledge of available testing services [41, 45–48]. In surveys of MSM in Germany, Malawi, and Swaziland, respondents cited shame and fear of stigma as major barriers to seeking testing [49–51]. Even once engaged in care, a comprehensive review of the literature in the United States identified mistrust of the medical system, lack of patient-provider race concordance, and negative provider attitudes toward bisexual behavior as specific factors in poor retention of black MSM in care [38]. Pathela and colleagues’ survey of men in New York City found heterosexual-identified MSM were also more likely to be of foreign origin or ethnic minority, suggesting that, perhaps due to conservative cultural backgrounds, they may be reluctant to identify same-sex behavior with homosexual orientation [47]. Further, the aforementioned ten-city survey among MSM in Brazil noted that HIV-related stigma acted as a potential barrier to care, though the impact of stigma associated with same-sex sexual behavior or orientations were not examined [3]. Thus further investigation is needed to understand how stigma against diverse sexualities translates into internalized homophobia (e.g. the acceptance of sexual stigma projected by others) and how it may inhibit the ability of individuals to access critical care. Future research drawing on frameworks that acknowledge and engage with the overlapping and complex intersection of race, class, gender and sexuality [52] will prove critical to unpacking these relationships.

Older age was also associated with increased odds for LP among MSM. With respect to age, our results were consistent with the findings of other studies focused on LP among MSM [29] among the general population [18, 19, 28, 30, 31, 53–55] and among a study of LP in Brazil [34]. Studies suggest that the progression of infection may be more rapid in older individuals [56, 57]. As documented in other studies among the general population, it is also possible that older age individuals are less likely to be tested for HIV because they themselves do not feel at risk, nor do their providers perceive them to be at risk for HIV infection [58, 59]. In Brazil, the Ministry of Health has drawn attention to HIV/AIDS among individuals beyond reproductive years by launching a campaign in 2010 and dedicating an entire AIDS Epidemiological Bulletin in 2011 to individuals over the age of 50 [60]. Despite this increased attention to the needs of older age individuals, the results of our analysis suggest that awareness of HIV, among both providers and older patients themselves, may be lacking. Therefore, efforts should be expanded to test older individuals and help those who test positive for HIV as they link to continued care.

Given the mounting literature documenting racial and ethnic disparities in linkage and retention in care [11], it was somewhat surprising that MSM of color did not have higher rates of LP. Since anti-discrimination laws have never been instituted in Brazil, it is commonly assumed that the country represents a genuinely integrated society, free from racial prejudice. Recently, however, several studies have documented a very different reality, collectively revealing that blacks, compared to whites, experience higher overall mortality [61], infant mortality [62], child mortality [63] and maternal mortality [64] while lacking access to quality postpartum services [65]. Racial disparities in many health outcomes are particularly stark in Salvador, where a recent meta-analysis of a nationally- representative sample found that black MSM were 3.4 times more likely to be HIV-positive than individuals identifying as white [66]. Further investigation is warranted to understand how race or ethnicity [67], especially as it is constructed in different cultural contexts, may differentially influence LP.

There are several limitations with this study. Mainly, the outcome measure used for LP focuses exclusively on CD4 count and therefore it remains unclear to which service the patient is presenting late. For example, depending on the organization of HIV/AIDS services, there are often several points at which an individual completes a CD4 evaluation: during testing, once initially enrolled in care, or upon initiating treatment [24]. Therefore by relying on CD4 count, we are unable to identify the specific gap in the provision of services. Also, while CD4 evaluation provides a consistent biomedical marker allowing for comparison across studies, this comparison must be made with caution as the reported prevalence of LP may differ based on when the CD4 evaluation was conducted. Further, due to the cross-sectional design of the study, retention in care over time could not be evaluated; we could not determinate causality, and results may also not be generalizable to other parts of Brazil.

Our study has several strengths. Importantly, this is the only study that exclusively focused on MSM and identified factors associated with LP in this population. Furthermore, it specified the sample based on sexual behaviors, rather than on self-reported sexual orientation. In so doing, we provided important insight into how behavioral factors influence LP among a population that is disproportionately impacted by the HIV/AIDS epidemic both globally and in Brazil. Finally, this is one of few articles focusing on LP outside of a high-income context.

## Conclusions

It is well known that men who self-identify as heterosexual may also have sex with men, and that people continue to have sex as they age, but recognizing that these characteristics are associated with late presentation to care raises challenges around a simple conceptualization of risk. While epidemiological evidence places young MSM at the center of transmission networks, the stigma around same-sex behaviors that translates to internal homophobia may prevent patients from seeking and staying engaged in HIV services. Similarly, the current focus of prevention and treatment efforts on younger age individuals may also limit conversations around safer sex practices with patients as they age. Developing effective strategies to reduce LP will require revisiting assumptions of who is at risk for HIV in order to make currently invisible people, visible.

## Abbreviations

LP: Late presentation
MSM: Men who have sex with men
HIV/AIDS: Human immunodeficiency virus infection/acquired immunodeficiency syndrome.
